# Race Detection and Density of *Rotylenchulus reniformis & Meloidogyne incognita* on Banana in Türkiye

**DOI:** 10.2478/jofnem-2025-0044

**Published:** 2025-11-17

**Authors:** Sümeyya Vuslat Dişkaya, İbrahim Halil Elekcioğlu

**Affiliations:** Faculty of Agriculture, Department of Plant Protection, Çukurova University, Adana 01330, Turkey

**Keywords:** greenhouse, morphology, plant parasitic nematodes, population dynamics, race detection

## Abstract

Plant-parasitic nematodes are responsible for substantial yield losses in banana-growing regions worldwide. In Türkiye, banana is traditionally grown in limited open-field areas, but greenhouse cultivation has rapidly expanded in recent years. However, information on nematodes associated with greenhouse bananas is extremely limited. This study aimed to determine the occurrence, races, and population densities of economically important nematodes in banana greenhouses. Surveys conducted between 2021–2022 in both new and old banana-producing regions revealed the presence of *Rotylenchulus reniformis* and *Meloidogyne incognita* in greenhouses in Mersin. *R. reniformis* was identified as race 2, while *M. incognita* was race 1. The population density of *M. incognita* increased when soil temperature rose above 10 °C, and *R. reniformis* densities increased above 20 °C. *M. incognita* was dominant during 2021–2022; however, its population was significantly suppressed by *R. reniformis* in 2022–2023.

Bananas are primarily cultivated in the tropical regions and are globally valued for their taste, nutritional content, and year-round availability (Khoozani et al., 2019). However, with advancements in cultivation techniques such as greenhouse production, banana cultivation has also expanded into subtropical and non-tropical regions, enabling production outside their traditional climatic zones. The total global banana production is reported to be approximately 135 million tons, with Türkiye ranking 26th worldwide by producing 997,000 tons (FAO, 2022). In Türkiye, bananas were previously grown in very limited open fields, but greenhouse cultivation has rapidly expanded in recent years. This shift is primarily attributable to the temperature limitations faced by banana production in non-tropical regions such as Türkiye. In response to abiotic stresses, greenhouse cultivation has expanded significantly, offering advantages such as improved yield under controlled environmental conditions and enhanced economic returns through continuous year-round production (Gubbuk et al., 2018; Lal Bahadur et al., 2020). Among the two most preferred banana cultivars for greenhouse production, the medium-height variety “Grand Nain” is prominent. In open-field cultivation, the most commonly grown banana variety is the short-height “Dwarf Cavendish” (Gubbuk et al., 2020).

Numerous disease agents and pests have been identified in banana-growing regions worldwide (Jones, 2009). Among these, plant-parasitic nematodes are particularly widespread and significant in the Mediterranean banana-growing regions (Bridge, 2000). The most significant plant-parasitic nematode species causing substantial yield losses in banana cultivation worldwide is *Rotylenchulus reniformis* (the reniform nematode) (Gowen and Quénéhervé, 1990; Brooks, 2004; Chávez and Araya, 2010). Furthermore, root-knot nematodes of the genus *Meloidogyne*, including *M. incognita*, *M. javanica*, and *M. arenaria*, are recognized as economically important species affecting banana production (Filipjev and Schuurmans Stekhoven, 1941; Sher and Allen, 1953; Elekcioğlu and Özarslandan, 2020). The presence of *M. incognita* and *M. javanica* was confirmed through data obtained from studies conducted in banana-producing regions of the Eastern Mediterranean Region (Elekcioğlu, 1992; Özarslandan and Elekcioğlu, 2010). *R. reniformis* was first reported in banana by Ayala and Roman (1963) in Puerto Rico. As semi-endoparasites, they are widely distributed and abundant in the soils of these crops (Robinson et al., 1997; Luc et al., 2005). *R. reniformis* appears to have the widest host range, with reports of breeding on the 315 plant species examined, including both cultivated plants and weeds (Luc et al., 2005). The symptoms caused by *R. reniformis* are relatively common, as are the damages caused by most plant-parasitic nematodes (Robinson et al., 1997; Crozzoli et al., 2004; Jones et al., 2013). These include stunted growth, poorly developed roots, and chlorosis (McSorley and Parrado, 1986; Bridge, 1988; Fogain and Gowen, 1997; Araya et al., 1999). There is a substantial body of literature on *M. incognita* infection in banana plantations (Jonathan and Rajendran, 2000; Kasapoğlu et al., 2015). However, the existing literature on the subject is limited to the interaction of *R. reniformis*. *Rotylenchulus macrosomus*, *Rotylenchulus parvus*, *M. incognita* (Elekcioğlu, 1992), and *Rotylenchulus borealis* (Akyazı et al., 2022) have been reported previously in T*ürkiye*. Elekcioğlu et al. (2024) conducted a survey in banana growing areas in Mersin in 2021–2022 and revealed the presence of *R. reniformis* for the first time at both the morphological and molecular levels.

This study aimed to examine the population dynamics of key nematode species in banana greenhouses and to obtain data relevant for control. Accordingly, the population trends of *R. reniformis* and *M. incognita* were monitored under greenhouse conditions in Kazanlı district of Mersin during the 2021–2023 growing seasons.

## Materials and Methods

### Soil sampling and nematode extraction

A survey was conducted in the Bozyazı, Kazanlı, and Silifke districts of Mersin province, where soil and root samples were collected from greenhouse-grown bananas. The greenhouses in the Kazanlı region of Mersin, which have been used for pepper cultivation for many years, have recently been renovated and repurposed for banana production. Considering the long-term cultivation of pepper in these greenhouses, their role as hosts for plant-parasitic nematodes, particularly root-knot nematodes (*Meloidogyne* spp.), is of significant concern during the renovation process. These nematodes also have a high potential to cause substantial yield losses in banana cultivation. In this study, the newly established banana plantations were included in the survey, and 10 greenhouses were examined, with soil samples collected from approximately 20–30 points in each greenhouse using a zigzag pattern. These samples were then blended, and 500 g of soil was taken for laboratory examination. Root samples were cut and crushed in a blender, and the soil samples were extracted using a modified Baermann funnel technique (Southey, 1986).

### Morphological identification

For the identification of *Meloidogyne* specimens, females were collected from roots and perineal patterns were mounted on slides (Xie, 2000). To prepare permanent slides, individuals belonging to the *Rotylenchulus* genus and second-stage juveniles of *Meloidogyne* specimens were killed in hot water, fixed at Triethanolamine-Formalin solution (14 ml formalin [40% formaldehyde] + 2 ml triethanolamine + 91 ml distilled water) (TAF) solution, and transferred to pure glycerin using the Seinhorst method (Seinhorst, 1959; Hooper, 1986). Samples were examined for morphological characters and morphometric measurements using an ocular micrometer, and ZEISS Scope A1 microscope equipped with an Axiocam 1cc5 camera. In total, 20 vermiform adult female and 10 male specimens of *R. reniformis* were obtained from soil samples collected from the greenhouse for morphological identification. The specimens were then prepared and evaluated under a light microscope in accordance with the morphological characters and morphometric measurements. In addition, female individuals were collected from the roots of the banana plant under binoculars and used for identification. For the identification of *R. reniformis* females in banana roots, root samples were stained using the acid fuchsin method following the protocol described by Byrd et al. (1983). Briefly, root segments were washed thoroughly, cleared in a 0.5% NaOCl solution for 2 min to remove pigments, and then rinsed with distilled water. The samples were subsequently immersed in 0.15% acid fuchsin solution and heated in a boiling water bath for 2 min to stain nematodes within the roots. After cooling, stained nematodes were then observed and counted under a stereomicroscope.

Additionally, *R. reniformis* specimens were examined using a scanning electron microscope (SEM) following the method of Eisenback (1985). Nematodes were transferred into 1 ml of 0.1 M phosphate buffer (pH 7.0) for 1 hr at 4°C prior to further processing (Seinhorst, 1959). Subsequently, 0.2–0.3 ml of 6% glutaraldehyde was added, and the samples were fixed at 4°C for 24 hr. To clean the nematode bodies, the same phosphate buffer was gently applied over them using a pipette, allowing thorough rinsing. After cleaning, the nematodes were immersed in 2% osmium tetroxide and incubated for 12 hr at 25°C. The osmium tetroxide solution was then replaced with fresh phosphate buffer five times at 15-min intervals. Following this, the nematodes were washed three times in 100% ethanol for 15 min each. Finally, the nematodes were placed on double-sided carbon conductive tape and coated with a 20-nm layer of gold using an automatic sputter coater. The prepared specimens were examined with an FEI Quanta FEG 650 SEM at the Central Research Laboratory of Çukurova University.

### Race detection of *R. reniformis* and *M. incognita*

The reproductive potential of *R. reniformis* was assessed using cowpea, castor, mustard, and cotton as differential hosts (Dasgupta and Seshadri, 1971). The reproductive potential of *M. incognita* was determined using tomato, pepper, tobacco, cotton, and peanut as different hosts (Rammah and Hirschmann, 1990; Robertson et al., 2009). Each pot used in the experiment had a width of 90 mm and a height of 85 mm, containing approximately 250 g of soil. Each test plant seedling was inoculated with 500 nematodes. The feeder roots of the seedlings were exposed by carefully removing the top layer of soil, allowing access to the roots. Subsequently, the requisite quantity of the nematode suspension was uniformly distributed around the exposed roots with the aid of a sterilized pipette. The experiment was set up with five replicates for each host plant. The plants were grown at an average temperature of 25 ± 1°C. After a 60-d inoculation period, plants were uprooted and *R. reniformis* races were classified based on the scheme proposed in Table 1. The nematode numbers were determined by extracting nematodes from 100 g of soil and by examining the entire root system of each host plant. The reproductive factor (Rf) of nematodes was calculated as Rf = Pf/Pi, where Pf represents the final population and Pi denotes the initial population in the pot (500 in the present experiments). The host with an average of ≤10 females and egg mass were classified as resistant (−) while those with >10 were classified as susceptible (+) (Rao and Ganguly, 1996). The Northern Carolina (NC) differential host test was used for the *Meloidogyne* species (Table 2). The standard differential host test uses egg mass ratings according to the following scale: 0 = 0 egg masses per plant, 1 = 1–2, 2 = 3–10, 3 = 11–30, 4 = 31–100 and 5 = >100. According to the NC differential host test (Hartman and Sasser, 1985), plants with average egg mass ratings ≤2 are classified as resistant and those with ratings >2 are classified as susceptible.

**Table 1:** The reaction of *Rotylenchulus reniformis* to the differential hosts (Rao and Ganguly, 1996).

**Races of *R. reniformis***	**Differential host plants**			
	**Castor**	**Cotton**	**Cowpea**	**Mustard**
Race 2	+	+	+	−
Race 4	+	+	+	+

**Table 2:** NC differential host test (Hartman and Sasser, 1985).

**Races of *M. incognita***	**Test plants**				
	**Tabaco**	**Cotton**	**Pepper**	**Tomato**	**Groundnut**
Race 1	−	−	+	+	−
Race 2	+	−	+	+	−
Race 3	−	+	+	+	−
Race 4	+	+	+	+	−
Race 5	−	−	−	+	−
Race 6	+	−	−	+	−

NC, Northern Carolina.

### Population dynamics of *R. reniformis* and *M. incognita*

*Rotylenchulus reniformis* and *M. incognita* were found in only one greenhouse in Kazanlı district of Mersin. This greenhouse, which was used to examine population fluctuations, covered approximately 0.4 ha and was divided into four blocks. From each block, soil samples were collected from 20 banana plants and from a depth of 30 cm using an auger. Nematodes were extracted using a modified Baermann funnel technique (Southey, 1986). To examine the population fluctuation of both species, soil samples were collected monthly between 2021 and 2023. The four blocks were sampled separately, and the results were averaged to determine the population levels.

## Results

### Survey results

As a result of the survey, in the Bozyazı and Silifke districts, *Helicotylenchus* spp. were detected either alone or in mixed populations with *Meloidogyne* spp., indicating varying nematode communities across these areas. By contrast, in the Kazanlı district, only *Meloidogyne* spp. were found, suggesting that root-knot nematodes were the predominant or sole plant-parasitic nematodes present there.

### Morphological and allometric features

#### Meloidogyne incognita

**Figure 1:**
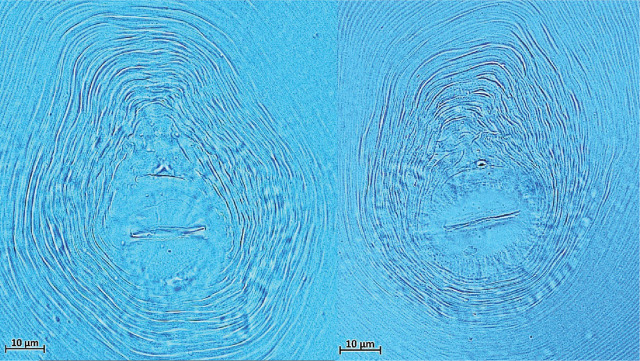
Perineal pattern of *Meloidogyne incognita*.

Females of *M. incognita* are pear-shaped, measuring 350–450 μm in length, and lack a posterior projection. The stylet is ca. 15.5 μm long. The perineal pattern in the vulval region is oval to rounded, typically exhibiting a high dorsal arch with wavy striation; the lateral field is absent or poorly defined. The tail measures 50–62 μm in length. Second-stage juveniles (J2) are 382–408 μm long, with a hyaline tail portion measuring 9–12 μm and a rounded tail tip. The morphological characteristics and allometrical measurements of the *M. incognita* specimens in this study were consistent with those described in previous studies (Jepson, 1987). Based on the morphological features of the perineal patterns in the vulval region, the nematodes collected from all banana greenhouses were identified as *M. incognita* (Fig. 1).

#### Rotylenchulus reniformis

**Figure 2:**
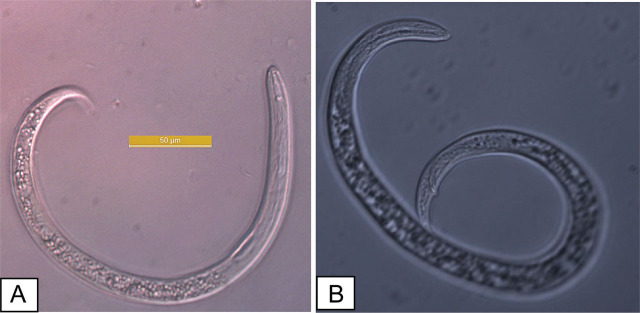
*Rotylenchulus reniformis*. Pre-adult female individual (A) and male individual (B).

**Figure 3:**
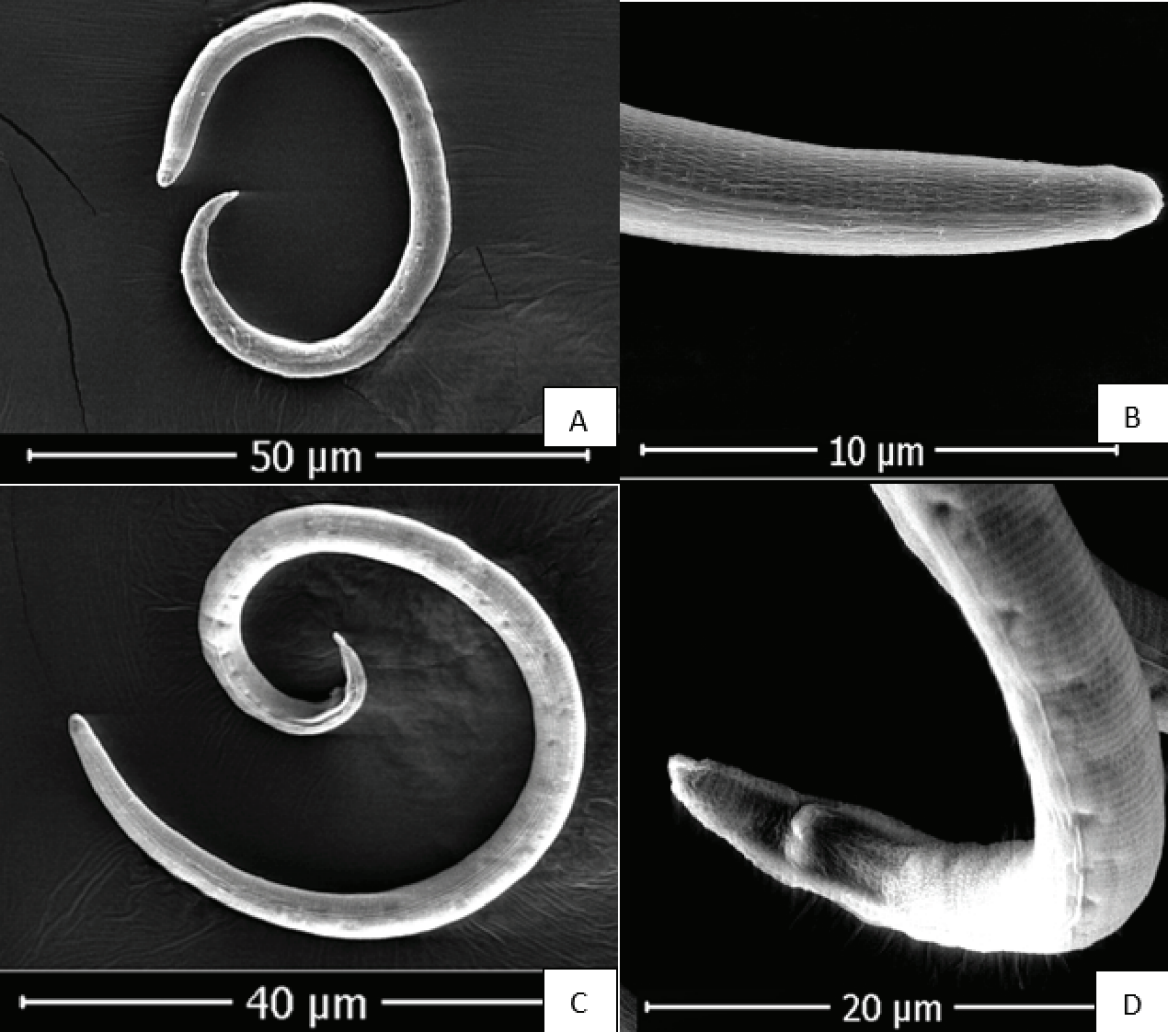
SEM photomicrographs of *Rotylenchulus reniformis*: (A) immature female, (B) anterior region of immature female, (C) adult male, and (D) tail region of male. SEM, scanning electron microscope.

The nematodes collected from banana greenhouses were identified as *R. reniformis* based on the morphological characteristics of the vermiform adult stage male and female individuals (Figs. 2A,B). The morphological characteristics and allometric measurements of the specimens examined in this study are consistent with those of *R. reniformis*, as previously described by Loof and Oostenbrink (1962), Dasgupta et al. (1968), Siddiqi (1972), and Germani (1978). *Immature female*: when killed by heat, immature females assume an open C-shaped posture. Their average body length ranges from 0.30 mm to 0.48 mm. The lateral field occupies slightly less than one-quarter of the body width. The lip region is elevated, conoid, rounded, and continuous with the body contour, bearing five annules. The stylet measures 17.5–25.2 μm in length, of moderate robustness, and ends in a small, rounded knob sloping posteriorly. The dorsal gland orifice is located more than halfway along the stylet in some individuals, approaching the stylet length in others. The metacorpus is elongate-oval with a prominent valve measuring approximately 4 μm (range: 4–6 μm). Esophageal glands overlap the intestine laterally and ventrally, with the ventral overlap being the most pronounced. The vulva is inconspicuous. The female reproductive system is amphidelphic, exhibiting two flexures in immature females and becoming highly convoluted in mature females. The tail typically measures more than twice the anal body diameter, conoid in shape, with a rounded terminus and a length of 28 μm (range: 26–35 μm). Phasmids appear as small pore-like structures located posterior to the anus, positioned at about the body width or slightly less. *Males*: the male species exhibit a more vermiform body compared with immature females. The lip region is high and rounded, but with weaker cephalic sclerotization and a more delicate stylet than that of immature females. The esophagus is markedly reduced, with the lumen often indistinct and the median bulb and valve poorly developed. Spicules are elongate, slender, and ventrally curved. Caudal alae are present but faint and do not extend fully to the tail tip. The lateral field in males, young females, and juveniles shows four non-areolated incisures. Males also possess a weak stylet, curved spicules, and a pointed tail, with a reduced esophagus. Immature females and males of *R. reniformis* were visualized using SEM (Fig. 3).

#### Race detection of R. reniformis and M. incognita

**Figure 4:**
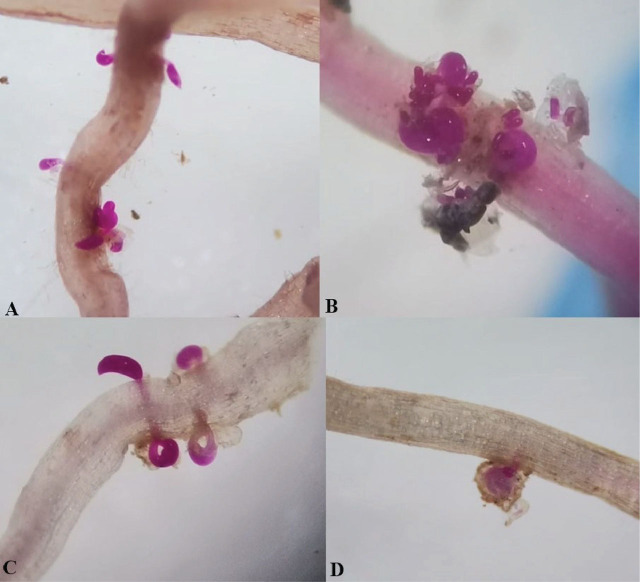
*Rotylenchulus reniformis* infecting root of castor (A), cowpea (B), and cotton (C,D) stained with acid fuchsin.

Differences were observed in the reproduction of *R. reniformis* on the host differential plants. Female individuals were observed in roots stained with acid fuchsin (Figs. 4A–D). *R. reniformis* reproduced on the three hosts (cotton, cowpea, and castor) but not on mustard. In the soil population, the rate of males to vermiform females was higher, while the rate of juveniles was similar. In consideration of the data obtained, *R. reniformis* was classified as race 2. This determination was based on the nematode's inability to reproduce on mustard (−), while females and egg mass developed well on cotton, castor, and cowpea (Table 3).

**Table 3:** Identification of races of *Rotylenchulus reniformis* infecting host differential plants.

**Host differential plants/number of nematodes**	**Soil + root (males/immature females/larvae)**	**Female/egg mass**
Cowpea	1,700/470/1,600	401/195
Cotton	760/380/540	270/88
Castor	840/380/870	387/208
Mustard	–	–

**Figure 5:**
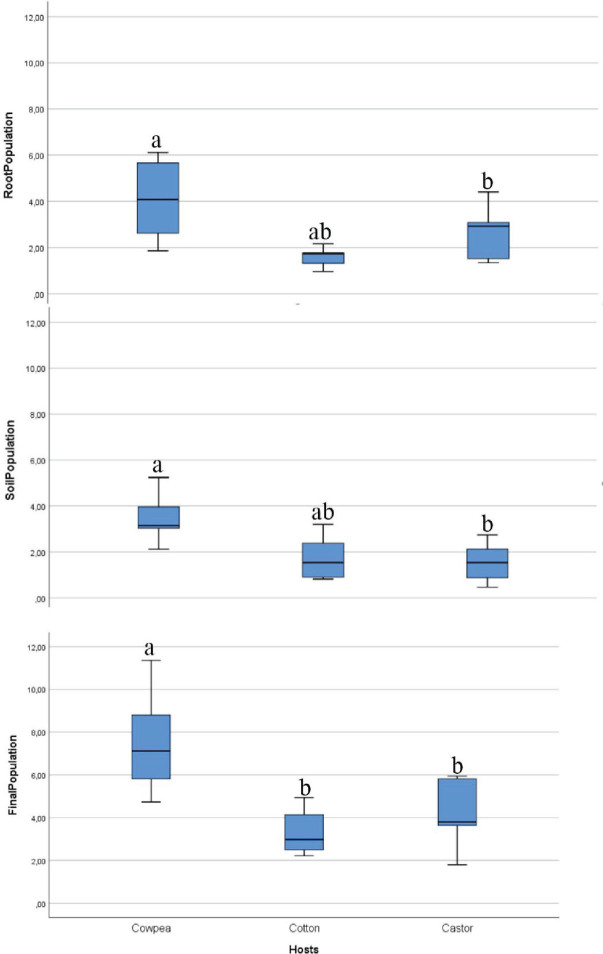
Reproduction of *Rotylenchulus reniformis* infecting cowpea, castor, and cotton. Error bars represent standard deviations. Different letters indicate significant differences at *P* ≤ 0.05 according to Tukey's test (*n* = 5).

The data presented in Figure 5 demonstrate that the population of *R. reniformis* was capable of penetrating, developing, and reproducing on cowpea, castor, and cotton, with notable variations in reproduction observed among the plants. The highest nematode populations were recorded in the roots and soil of cowpea, resulting in the largest population increase among the three host plants evaluated, with a 7.5-fold multiplication. By contrast, the development and reproduction of *R. reniformis* on cotton were significantly lower than on cowpea, with only a 3.3-fold increase observed. The results indicated that cowpea was the most favorable host, followed by cotton and castor. Nevertheless, the nematode was able to reproduce on all three hosts.

Reproduction differences of *M. incognita* were observed among the differential host plants. *M. incognita* reproduced on the four host plants, with the exception of peanut. The lowest number of J2 was observed in the roots and soil of cotton and tobacco, while the highest number of J2 was recorded in tomato and pepper. Based on the data obtained, *M. incognita* was identified as race 1 (Table 4).

**Table 4:** Identification of races of *Meloidogyne incognita* according to final population (Rf), 0–5 egg mass reaction scale, root-gall index (0–10).

	Tobacco Mean ± SE^**^	**Cotton Mean ± SE**	**Tomato Mean ± SE**	**Pepper Mean ± SE**	**Groundnut Mean ± SE**
Soil + root final population (Rf)	0.6 ± 0.13 bc^*^	0.62 ± 0.22 bc	2.9 ± 0.86 a	2.3 ± 0.29 ab	0 ± 0 c
Egg masses	2.4 ± 0.2 c	2.6 ± 0.2 bc	3.6 ± 0.2 a	3.4 ± 0.2 ab	0 ± 0 d
Root-Gall Index	1.2 ± 0.2 b	1.2 ± 0.2 b	4.2 ± 0.2 a	3.4 ± 0.2 a	0 ± 0 c

* Values containing the same letter in the same row are not different from each other, *P* < 0.05 (TUKEY Test).

** Values are means ± S.E. of five replicates.

SE, standard error.

The population of *M. incognita* was successfully penetrated, developed, and reproduced on tobacco, tomato, pepper and cotton, but notable variations in reproduction were observed among the plants. The highest nematode populations were recorded in tomato and pepper plants with final population reproduction rates of 2.9 and 2.3, respectively, among the four host plants evaluated. By contrast, *M. incognita* exhibited limited development and reproduction on cotton and tobacco, with only 0.62- and 0.6-fold increases, respectively. These findings suggest that tomato and pepper are the most favorable hosts for *M. incognita* while cotton and tobacco were classified as poor hosts.

#### Population dynamics of R. reniformis and M. incognita

**Figure 6:**
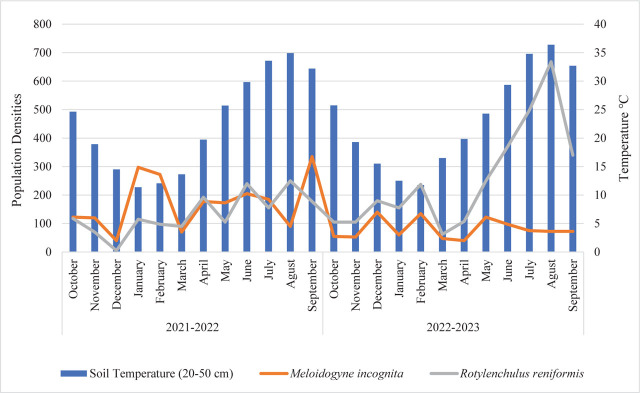
Population density of *Rotylenchulus reniformis* and *Meloidogyne incognita* on bananas in the greenhouse.

The banana plants in the experimental greenhouse were approximately 8 years old. *M. incognita* and *R. reniformis*, the major nematode in the area, coexisted as a mixed population in the rhizosphere between 2021 and 2023 (Fig. 6). The *M. incognita* population densities showed three distinct peaks in 2022: 297 J2/100 g soil in January, 272 in June, and 335 in September 2022. However, it was observed that the densities declined to 140, 135, and 122 J2/100 g soil, respectively, in December 2022, February, and May 2023. The lowest population densities of *M. incognita* were observed in December 2021 and January 2023, with 50 J2/100 g soil present in each instance.

In the greenhouse, the population density of *R. reniformis* was observed to reach four peaks, with 117, 192, 240, and 250 vermiform life stages nematode/100 g soil, respectively, in October 2021 and April, June and July 2022. The following year, three additional peaks were observed in December 2022, February, and August 2023, with densities of 180, 237, and 668 vermiform life stages nematode/100 g soil, respectively. Subsequently, the number of nematodes exhibited a marked increase from April to August 2023, reaching a density of 668 vermiform life stages nematode/100 g soil. The highest population density of *R. reniformis* coincided with a soil temperature of 35°C in both years. Conversely, *R. reniformis* exhibited the lowest population density, with only five vermiform life stages nematode/100 g soil in December 2021. The lowest population density between 2022 and 2023 was observed in March, with a count of 62 vermiform life stages nematode/100 g soil. These findings suggest that *R. reniformis* was newly introduced into this greenhouse in 2021 and that its population level increased rapidly in the following years. Results from the two-seasons study revealed *M. incognita* initially exhibited higher population densities than *R. reniformis*. As demonstrated in Figure 6, the population density of *M. incognita* was approximately three times higher than *R. reniformis* in January and February. However, the population growth rate of *R. reniformis* matched that of *M. incognita* in April, doubled in May, and continued to rise, reaching nine-fold levels in August. In conclusion, *R. reniformis* showed a population increase in 2023, whereas *M. incognita* was suppressed.

## Discussion

Previous studies conducted in banana plantations in Türkiye over the past 30–35 years have shown that the *Meloidogyne* species are commonly found (Elekcioğlu, 1992; Özarslandan and Elekcioğlu, 2010; Kasapoğlu et al., 2015). More recently, Elekcioğlu et al. (2024) reported for the first time the presence of *R. reniformis* in banana fields in Türkiye. *R. reniformis* was classified as race 2, while *M. incognita* was identified as race 1. The current literature on the occurrence and distribution of reniform nematode races is inadequate. The first detection of this nematode in Türkiye was in 2024, yet no race study was conducted. However, numerous studies show instances of the persistence of diverse races of reniform nematodes on a range of crops cultivated globally (Dasgupta and Seshadri, 1971; Rao and Ganguly, 1996; Singh and Azam, 2011). Rao and Ganguly (1996) reported that an *R. reniformis* race 2 population was pathogenic on cowpea, cotton, and castor. Similar findings were reported by Adam et al. (2018) in Egypt. Vadhera et al. (1999) observed a distinct population of reniform nematodes that were unable to reproduce on *Ricinus communis* in Jabalpur, India. Previous race identification studies in Türkiye have reported *M. incognita* as race 1, race 2, race 3, and race 4 (Söğüt and Elekcioğlu, 2000; Mennan and Ecevit, 2001; Devran and Söğüt, 2011). Gürkan and Çetintaş (2024) determined that the *M. incognita* was race 1 according to the NC differential host test. Furthermore, Gürkan et al. (2019) detected race 1, race 2, and race 3 of *M. incognita* in 20 populations examined in the Mediterranean region.

On the contrary, population densities of plant-parasitic nematodes were monitored in a banana greenhouse, where it was found that only *M. incognita* was present among root-knot nematodes. This may be linked to the historical cultivation of peppers in the area. In the greenhouse where the experiment was conducted, *R. reniformis* coexisted with an annual average population density of approximately 1,615 individuals (juveniles and vermiform males/females) alongside *M. incognita* with about 2,088 second-stage juveniles (J2) during 2021–2022. In 2022–2023, *R. reniformis* populations increased to around 2,828 individuals, while *M. incognita* populations decreased to approximately 970 J2. While *M. incognita* was the dominant species in the first year, a notable suppression of root-knot nematodes by *R. reniformis* was observed in the following year. A similar study in banana fields identified *Meloidogyne* spp. as the most dominant nematode species, accounting for 88% of the nematode population (Gaidashova et al., 2004). Additionally, population monitoring of *R. reniformis* showed the highest densities in August and the lowest in October (Gantait et al., 2006), consistent with our findings. Previous research also suggests that fluctuations in nematode populations are influenced by soil temperature (Kasapoğlu et al., 2015; Evlice et al., 2022), with significant population changes occurring as temperatures rise, likely due to interspecific competition. Moreover, monoculture practices typical of banana cultivation provide a continuous food source for plant-parasitic nematodes, promoting consistently high populations. Even when overall nematode densities remain low, their feeding activity can still negatively impact seedling development. Banana plants produce fruit year-round while simultaneously generating new shoots. Because the first seedlings emerge in close proximity to the mother plant, nematodes can easily colonize and feed on these new seedlings once the mother plant is cut down. This feeding damage impairs the growth and vigor of the young plants, leading to weak development, lighter bunches, and smaller, lower-quality banana fruits (Özarslandan, 2019). However, significant reductions in nematode populations have been observed in seedlings planted at greater distances from the mother plant (Gowen and Quénéhervé, 1990).

The results of this study indicate that the population of *M. incognita* begins to increase in January in the Kazanlı district of Mersin province. Due to the occurrence of *R. reniformis* in banana fields, internal quarantine measures should be implemented to prevent the spread of this nematode within the country. If quarantine measures are not implemented and *Rotylenchulus* spreads, this could lead to severe infestations that damage root systems and cause substantial yield losses. Consequently, growers would face significant economic impacts due to decreased productivity and increased management costs. *R. reniformis* has been reported to cause significant yield losses ranging from 25% to 60% at different population levels (Robinson et al., 1997; Crozzoli et al., 2004; Jones et al., 2013). Furthermore, additional survey studies should be conducted to determine whether this nematode is present in other banana fields within the country. Given that *R. reniformis* has a diverse range of hosts, including cotton, which is a major host and causes significant damage, the importance of quarantine methods increases. It is therefore important to ensure that the production material is clean and certified.

The results of this study may serve as a foundation for future research on the population dynamics of the reniform nematode (*R. reniformis*) and the root-knot nematode (*M. incognita*), providing essential information for effective nematode management in banana cultivation. A detailed understanding of their seasonal population fluctuations, reproduction rates, and growth dynamics can enable growers and researchers to better time control measures, target treatments at the most susceptible nematode life stages, and minimize unnecessary pesticide applications. In particular, applying plant-based extracts—environmentally friendly alternatives to chemical pesticides—during periods of low nematode density may increase treatment efficacy and improve the overall success of integrated pest management strategies. Further research can be carried out to determine the efficacy of existing resistant cultivars under Turkish conditions. However, the fact that *R. reniformis* does not produce a typical symptom on the roots like root-knot nematodes may cause this pest to be overlooked and quarantine measures may not be sufficient. Consequently, it is advised that a survey encompassing the entire region be conducted to ascertain the presence of *R. reniformis* in greenhouses and that quarantine measures be implemented. In addition, understanding the population dynamics of these two nematodes under field conditions may facilitate the selection and timing of control methods.

## References

[j_jofnem-2025-0044_ref_001] Adam M., Diab S. F., Farahat A., Alsayed A. A., Heuer H. (2018). Molecular identification, race detection, and life cycle of *Rotylenchulus reniformis* in Egypt. Nematropica.

[j_jofnem-2025-0044_ref_002] Akyazı F., Güvercin B., Yılmaz O. (2022). Morphological and molecular characterization of *Rotylenchulus borealis* Loof and Oostenbrink, 1962 from Turkey. Turkish Journal of Agricultural and Natural Sciences.

[j_jofnem-2025-0044_ref_003] Araya M., Vargas A., Cheves A. (1999). Nematode distribution in roots of banana (Musa AAA cv. Valery) in relation to plant height, distance from the pseudostem and soil depth. Nematology.

[j_jofnem-2025-0044_ref_004] Ayala A., Roman J. (1963). Distribution and host range of the burrowing nematode in Puerto Rican soils. Journal of Agriculture.

[j_jofnem-2025-0044_ref_005] Bridge J. (1988). Plant parasitic nematode problems in the Pacific Islands. Journal of Nematology.

[j_jofnem-2025-0044_ref_006] Bridge J. (2000). Nematodes of bananas and plantains in Africa: Research trends and management strategies relating to the small-scale farmer. Acta Horticulturae.

[j_jofnem-2025-0044_ref_007] Brooks F. E. (2004). Plant-parasitic nematodes of banana in American Samoa. Nematropica.

[j_jofnem-2025-0044_ref_008] Byrd D. W., Kirkpatrick T., Barker K. R. (1983). An improved technique for clearing and staining plant tissues for detection of nematodes. Journal of Nematology.

[j_jofnem-2025-0044_ref_009] Chavez C., Araya M. (2010). Spatial-temporal distribution of plant-parasitic nematodes in banana (*Musa* AAA) plantations in Ecuador. Journal of Applied Biosciences.

[j_jofnem-2025-0044_ref_010] Crozzoli R., Perichi G., Vovlas N., Greco N. (2004). Effect of *Rotylenchulus reniformis* on the growth of papaya in pots. Nematropica.

[j_jofnem-2025-0044_ref_011] Dasgupta D. R., Seshadri A. R. (1971). Races of the reniform nematode, *Rotylenchulus reniformis* Linford and Oliveira, 1940. Indian Journal of Nematology.

[j_jofnem-2025-0044_ref_012] Dasgupta D. R., Raski D. J., Sher S. A. (1968). A revision of the genus Rotylenchulus Linford and Oliveira, 1940 (Nematoda: Tylenchidae). Proceedings of the Helminthological Society of Washington.

[j_jofnem-2025-0044_ref_013] Devran Z., Söğüt M. A. (2011). Characterizing races of *Meloidogyne incognita*, *M. javanica* and *M. arenaria* in the West Mediterranean region of Turkey. Crop Protection.

[j_jofnem-2025-0044_ref_014] Eisenbach J., Southey J. F. (1985). Techniques for preparing nematodes for scanning electron microscopy. Laboratory methods for work with plant and soil nematodes.

[j_jofnem-2025-0044_ref_015] Elekcioğlu I. H. (1992). Untersuchungen zum Auftreten und zur Verbreitung phytoparasitaerer Nematoden in den Landwirtschaftlichen Hauptkulturen des Ostmediterranen Gebietes der Türkei.

[j_jofnem-2025-0044_ref_016] Elekcioğlu I. H., Özarslandan A. (2020). Zararlı nematodlar ve mücadele yolları. Tarım Gündem Dergisi Özel Yayını.

[j_jofnem-2025-0044_ref_017] Elekcioğlu I. H., Uludamar E. B. K., Dişkaya S. V., Avcıoğlu S., Çağlar B. K. (2024). Characterization of *Rotylenchulus reniformis* Linford and Oliveira, 1940 (Tylenchida: Hoplolaimidae) in a banana greenhouse in Turkey. Crop Protection.

[j_jofnem-2025-0044_ref_018] Evlice E., Toktay H., Yatkın G., Erdoğuş F. D., İmren M. (2022). Population fluctuations of root knot nematodes *Meloidogyne chitwoodi* and *M. hapla* under field conditions. Phytoparasitica.

[j_jofnem-2025-0044_ref_019] FAO (2022). https://www.fao.org/statistics/en/.

[j_jofnem-2025-0044_ref_020] Filipjev I., Stekhoven J. S. (1941). A manual of agricultural helminthology.

[j_jofnem-2025-0044_ref_021] Fogain R., Gowen S. R. (1997). Damage to roots of Musa cultivars by *Radopholus similis* with and without protection of nematicides. Nematropica.

[j_jofnem-2025-0044_ref_022] Gaidashova S. V., Okech S., van den Berg E., Marais M., Gatarayiha C. M., Ragama P. E. (2004). Plant-parasitic nematodes in banana-based farming systems in Rwanda: Species profile, distribution and abundance. African Plant Protection.

[j_jofnem-2025-0044_ref_023] Gantait V. V., Bhattacharya T., Chatterjee A. (2006). Fluctuation of nematode populations associated with banana plantation in Medinipur District, West Bengal, India. Indian Journal of Nematology.

[j_jofnem-2025-0044_ref_024] Germani G. (1978). Caractères morpho-biométricques de trois espèces oust-africaines de *Rotylenchulus* Linford & Oliveira, 1940 (Nematoda: Tylenchida). Revue de Nématologie.

[j_jofnem-2025-0044_ref_025] Gowen S. R., Queneherve P., Luc M., Sikora R. A., Bridge J. (1990). Nematode parasites of bananas, plantains and abaca. Plant Parasitic nematodes in subtropical and tropical Agriculture.

[j_jofnem-2025-0044_ref_026] Gubbuk H., Bakry F., Guven D., Taskiran O., Mathieu Y. (2020). Agronomic evaluation of new Cavendish banana cultivars for growing in different protected cultivation areas in Turkey. Acta Horticulturae.

[j_jofnem-2025-0044_ref_027] Gubbuk H., Gunes E., Guven D. (2018). Comparison of open-field and protected banana cultivation for some morphological and yield features under subtropical conditions. Acta Horticulturae.

[j_jofnem-2025-0044_ref_028] Gürkan T., Çetintaş R. (2024). Detection of root-knot nematode species and races in Kahramanmaraş Province, Türkiye. Kahramanmaraş Sütçü İmam Üniversitesi Tarım Ve Doğa Dergisi.

[j_jofnem-2025-0044_ref_029] Gürkan B., Çetintaş R., Gürkan T. (2019). Gaziantep ve Osmaniye Sebze Alanlarında Bulunan Kökur Nematodu Türleri (*Meloidogyne* spp.)'nin Teşhisi ile Bazı Nematod Popülasyon Irklarının Belirlenmesi. Kahramanmaraş Sütçü İmam Üniversitesi Tarım ve Doğa Dergisi.

[j_jofnem-2025-0044_ref_030] Hartman K. M., Sasser J. N., Barker K. R., Carter C. C., Coop Sasser JN (1985). Identification of *Meloidogyne* species by differential host test and perineal pattern morphology. An advanced treatise on *Meloidogyne*.

[j_jofnem-2025-0044_ref_031] Hooper D. J., Southey J. F. (1986). Extraction of free-living stages from soil. Laboratory methods for work with plant and soil nematodes.

[j_jofnem-2025-0044_ref_032] Jepson S. B. (1987). Identification of root-knot nematodes.

[j_jofnem-2025-0044_ref_033] Jonathan E. I., Rajendran G. (2000). Assessment of avoidable yield loss in banana due to root-knot nematode *Meloidogyne incognita*. Indian Journal of Nematology.

[j_jofnem-2025-0044_ref_034] Jones D. R. (2009). Disease and pest constraints to banana production. Acta Horticulturae.

[j_jofnem-2025-0044_ref_035] Jones J. T., Haegeman A., Danchin E. G., Gaur H. S., Helder J., Jones M. G., Kikuchi T., Manzanilla-López R., Palomares-Rius J. E., Wesemael W. M., Perry R. N. (2013). Top 10 plant-parasitic nematodes in molecular plant pathology. Molecular Plant Pathology.

[j_jofnem-2025-0044_ref_036] Kasapoğlu E. B., Yoraz G., Elekcioğlu İH. (2015). Investigation on population dynamics of important plant parasitic nematodes (*Helicotylenchus multicinctus*, *H. dihystera*, and *Meloidogyne* spp.) (Nemata) in banana greenhouses grown in Bozyazi (Mersin). Turkish Journal of Entomology.

[j_jofnem-2025-0044_ref_037] Khoozani A. A., Birch J., Bekhit A. E. D. A. (2019). Production, application and health effects of banana pulp and peel flour in the food industry. Journal of Food Science and Technology.

[j_jofnem-2025-0044_ref_038] Lal Bahadur A., Singh D., Singh S. K. (2020). A review on successful protected cultivation of banana (Musa). Plant Archives.

[j_jofnem-2025-0044_ref_039] Loof P. A. A., Oostenbrink M. (1962). *Rotylenchulus borealis* N. Sp., with a key to the species of *Rotylenchulus*. Nematologica.

[j_jofnem-2025-0044_ref_040] Luc M., Sikora R., Bridge J. (2005). Plant Parasitic Nematodes in subtropical and tropical agriculture.

[j_jofnem-2025-0044_ref_041] McSorley R., Parrado J. L. (1986). *Helicotylenchus multicinctus* on bananas: An international problem. Nematropica.

[j_jofnem-2025-0044_ref_042] Mennan S., Ecevit O. (2001). Bafra ve Çarşamba Ovaları'ndan elde edilen bazı *Meloidogyne incognita* (Kofoid and White, 1919) (Nemata: Heteroderidae) popülasyonlarında ırk tespiti. Türkiye Entomoloji Dergisi.

[j_jofnem-2025-0044_ref_043] Özarslandan A. (2019). New approaches for sucker selection in greenhouse banana to reduce nematode number in subtropics. Indian Journal of Horticulture.

[j_jofnem-2025-0044_ref_044] Özarslandan A., Elekcioğlu İ.H. (2010). Identification of the root-knot nematode species (*Meloidogyne* spp.) (Nemata: *Meloidogynidae*) collected from different parts of Turkey by molecular and morphological methods. Türkiye Entomoloji Dergisi.

[j_jofnem-2025-0044_ref_045] Rammah A., Hirschmann H. (1990). Morphological comparison of three host races of *Meloidogyne javanica*. Journal of Nematology.

[j_jofnem-2025-0044_ref_046] Rao G. M. V. P., Ganguly S. (1996). Host preference of six geographical isolates of reniform nematode, *Rotylenchulus reniformis*. Indian Journal of Nematology.

[j_jofnem-2025-0044_ref_047] Robertson L., Diez-Rojo M. A., Lopez-Perez J. A., Piedra Buena A., Escuer M., Lopez Cepero J., Martinez C., Bello A. (2009). New host races of *Meloidogyne arenaria*, *M. incognita*, and *M. javanica* from horticultural regions of Spain. Plant Disease.

[j_jofnem-2025-0044_ref_048] Robinson A. F., Inserra R. N., Caswell-Chen E. P., Vovlas N., Troccoli A. (1997). *Rotylenchulus* species: Identification, distribution, host ranges and crop plant resistance. Nematropica.

[j_jofnem-2025-0044_ref_049] Seinhorst J. W. (1959). A rapid method for the transfer of nematodes from fixative to anhydrous glycerin. Nematologica.

[j_jofnem-2025-0044_ref_050] Sher S. A., Allen M. W. (1953). Revision of the genus *Pratylenchus* (Nematoda: Tylenchidae). University of California Publications in Zoology.

[j_jofnem-2025-0044_ref_051] Siddiqi M. R. (1972). Rotylenchulus reniformis. C.I.H. Descriptions of Plant-Parasitic Nematodes.

[j_jofnem-2025-0044_ref_052] Singh N., Azam M. F. (2011). Studies on the pathogenecity of three host races of *Rotylenchulus reniformis* on castor. Archives of Phytopathology and Plant Protection.

[j_jofnem-2025-0044_ref_053] Söğüt M., Elekcioğlu I. H. (2000). Determination of *Meloidogyne* Goeldi, 1892 (Nemata: Heteroderidae) species races found in vegetable growing areas of the Mediterranean region of Turkey. Turkish Journal of Entomology.

[j_jofnem-2025-0044_ref_054] Southey J. F. (1986). Laboratory methods for work with plant and soil nematodes.

[j_jofnem-2025-0044_ref_055] Vadhera I., Bhatt J., Shukla B. N. (1999). Prevalence of a new race of *Rotylenchulus reniformis* in Madhya Pradesh. Journal of Mycological Plant Pathology.

[j_jofnem-2025-0044_ref_056] Xie H. (2000). Taxonomy of plant nematodes.

